# Microstructural Analysis of Sand Reinforced by EICP Combined with Glutinous Rice Slurry Based on CT Scanning

**DOI:** 10.3390/ma18071563

**Published:** 2025-03-30

**Authors:** Jianye Wang, Xiao Li, Liyun Peng, Jin Zhang, Shuang Lu, Xintao Du

**Affiliations:** 1 School of Civil and Transportation Engineering, Beijing University of Civil Engineering and Architecture, Beijing 100044, China; wangjianye@bucea.edu.cn (J.W.); 19801305720@163.com (X.L.); zj18152160456@163.com (J.Z.); 2108590124014@stu.bucea.edu.cn (S.L.); 2 Beijing Institute of Engineering Geology, Beijing 100037, China; dxt13934310093@163.com

**Keywords:** glutinous rice slurry, EICP, unconfined compressive strength, permeability, microstructural analysis, micro-computed tomography

## Abstract

Sandy soils are prone to engineering issues due to their high permeability and low cohesion in the natural environment. Therefore, eco-friendly reinforcement techniques are required for projects such as subgrade filling and soft soil foundation reinforcement to enhance their performance. This study proposes a synergistic reinforcement method that combines Enzyme-Induced Calcium Carbonate Precipitation with Glutinous rice slurry (G-EICP). The macroscopic mechanical properties and pore structure evolution of reinforced sand were systematically investigated through triaxial permeability tests, unconfined compressive strength (UCS) tests, and microstructural characterization based on Scanning Electron Microscope (SEM) and Micro- Computed Tomography (CT) tests. The results indicate that when the glutinous rice slurry volume ratio (*V*_G_) reaches 10%, the UCS of G-EICP-reinforced soil peaks at 449.2 kPa. The permeability coefficient decreases significantly with increasing relative density (*D*_r_), *V*_G_, confining pressure (*σ*_3_), and seepage pressure (*p*). Microstructural analysis reveals that glutinous rice slurry may promote calcium carbonate crystal growth, potentially by providing nucleation sites, establishing a dual mechanism of skeleton enhancement and pore-throat clogging. The increased incorporation of glutinous rice slurry reduces the number of connected pores, lowers the coordination number, and elevates tortuosity, thereby inducing marked enhancements in both the strength and permeability of the treated soil compared to plain soil.

## 1. Introduction

Sandy soil, as a typical porous medium, is frequently confronted with structural instability and seepage failure in engineering applications due to its high permeability and cohesionless [1,2]. Statistics indicate that the area affected by desertification accounts for approximately 35% of the global land area [3]. Consequently, optimizing the structural stability and anti-seepage performance of sandy soil through rational reinforcement has become a critical challenge in geotechnical engineering [4].

Enzyme-induced calcium carbonate precipitation (EICP), an emerging eco-friendly soil stabilization technique, has garnered widespread attention owing to its lower energy consumption and reduced environmental contamination compared to conventional methods such as lime/cement stabilization or dynamic compaction [5,6]. This technology has already been proven to be feasible in applications such as tunnel engineering [7]. This technique employs urease to catalyze urea hydrolysis, generating carbonate ions that combine with calcium ions to form calcium carbonate crystals. These crystals establish cementitious structures and fill interparticle pores, thereby significantly enhancing the soil mechanical properties [8]. Experimental studies have demonstrated that EICP can reduce soil permeability and improve its strength. For instance, Khan et al. [9] observed a progressive decline in silty sand permeability with increasing EICP solution concentration, with the highest permeability decline of 75%; Zango et al. [10] reported that the unconfined compressive strength of residual soil reinforced with EICP could be increased by up to 275%. However, the practical applications of EICP still face limitations: insufficient nucleation sites lead to the formation of small-sized calcite crystals, which fail to effectively fill medium-to-large pores in coarse-grained soils. This results in discontinuous cementation networks and suboptimal reinforcement efficiency [11,12], ultimately constraining the technique’s effectiveness in improving the strength and anti-seepage performance [13].

To address these challenges, recent studies have explored synergistic combinations of EICP with supplementary materials. For example, Jain. S et al. [14] demonstrated that adding 3 g/L of xanthan gum to EICP-reinforced soil can effectively enhance the strength and reduce the air-entry value of reinforced soil. Lemboye et al. [15] combined EICP with sodium alginate and guar gum to form viscous gels that effectively clog soil pores and reduce permeability. Singh et al. [16] found that xanthan gum generated water-insoluble gel-like precipitates in EICP solutions, further strengthening the soil matrix. Another study [17] revealed that the addition of 2272 g skimmed milk powder per cubic meter of soil can provide additional nucleation sites, promoting calcium carbonate crystal growth and precipitation to enhance soil strength. Despite these advances, existing composite materials often suffer from high costs or complex production processes [18,19], hindering their large-scale application.

In contrast to synthetic additives, glutinous rice slurry, a traditional Chinese construction material for reinforcing city walls, was the earliest record in the ancient building manual “Tian Gong Kai Wu”, written during the Ming Dynasty (1368–1644 AD) [20]. It offers distinct advantages due to its inherent adhesiveness, cost-effectiveness, and environmental compatibility, and has been extensively utilized in historical architecture and soil stabilization [21,22]. Previous studies have demonstrated through grouting methods that a small amount of glutinous rice slurry can enhance the efficiency of MICP-reinforced soil [23,24]. However, there is currently a lack of research on the effects of reinforcing sandy soil with glutinous rice slurry in combination with EICP, especially studies based on the mixing method. Additionally, there is still a deficiency in the analysis of the three-dimensional pore structure of soil reinforced using this combined method. Leveraging these eco-friendly attributes and superior reinforcement potential, this study proposes a novel glutinous rice slurry-EICP (G-EICP) synergistic stabilization system to improve the strength and anti-seepage performance of sandy soil. Traditional reinforcement techniques (i.e., cement) contribute to 8% of anthropogenic CO₂ emissions, exacerbating climate degradation, such as the greenhouse effect and global warming [25]; hence, novel reinforcement techniques hold significant value.

Through a series of laboratory experiments, this study investigates the influence of soil relative density and glutinous rice slurry volumetric content on the unconfined compressive strength (UCS) of treated sand, while analyzing the combined effects of these variables, confining pressure, and seepage pressure on permeability. Furthermore, by integrating scanning electron microscopy (SEM) and micro-computed tomography (micro-CT) results, the “glutinous rice slurry–calcium carbonate” synergistic reinforcement mechanism is elucidated at the microscale, with particular emphasis on the relationship between pore structure evolution and macroscopic mechanical behavior.

## 2. Materials and Methods

### 2.1. Materials

#### 2.1.1. Soil

**Figure 1:**
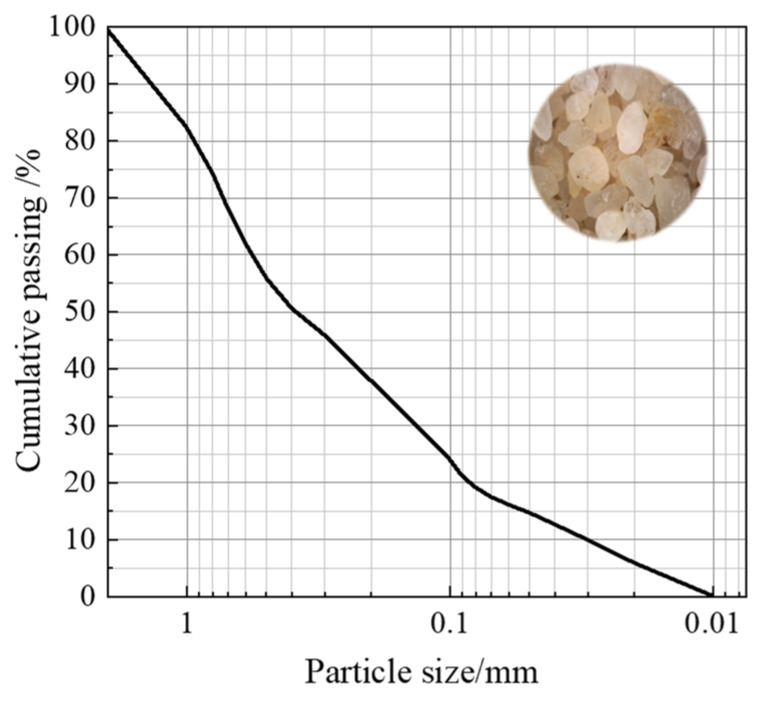
The particle size distribution curve of sand used in this study.

The sand used in this study was ISO-standard Fujian sand (Xiamen, China) with a particle size distribution of 0.075–2.0 mm. Following the Test Methods of Soils for Highway Engineering (JTG 3430-2020) [26], the maximum and minimum void ratios were determined as *e*_max_ = 0.654 and *e*_min_ = 0.325, respectively. The specific gravity (*G*_s_) was measured as 2.6, and the uniformity coefficient (*C*_u_) was calculated as 36.69. The particle size distribution curve is illustrated in Figure 1.

#### 2.1.2. EICP Solution

The EICP cementation solution was prepared by first dissolving analytical-grade urea (CO(NH_2_)_2_) and calcium chloride (CaCl_2_) (Xilong, Shantou, China)in distilled water to form separate solutions, which were then mixed at a 1:1 volumetric ratio. The concentration of the solution was set to 1 mol/L. The urease enzyme was extracted from commercially available soybeans, exhibiting an enzymatic activity of 6.888 mM urea/min, as measured using a conductivity meter (Brand and model: DDSJ-308A, China). The extraction procedure included the following steps: (1) drying, grinding, and sieving the soybean grains; (2) mixing the soybean powder with deionized water, followed by centrifugation at 3000 rpm for 30 min; and (3) collecting the supernatant, which was stored at 4 °C for subsequent use.

#### 2.1.3. Glutinous Rice Slurry

Glutinous rice slurry was synthesized by heating glutinous rice flour in a water bath. Specifically, the preparation involved: (1) blending glutinous rice flour with deionized water; (2) heating the mixture at 90 °C for 1 h under continuous stirring, with periodic water replenishment to maintain a constant liquid level; and (3) cooling the slurry to room temperature. To ensure the flowability and homogeneity of the glutinous rice slurry, the authors prepared samples with varying concentrations using the aforementioned method and found that when the glutinous rice content exceeded 12%, the mixture failed to form a homogeneous slurry. Therefore, the concentration (*C*_G_) was fixed at 12%. The viscosity of the slurry measured with a rotational viscometer ((Brand and model: SN-NDJ-4, China) at 25 °C and 30 rpm was *η* = 5.9295 × 10^4^ mPa⋅s.

### 2.2. Specimen Preparation

Existing relevant studies [23,24] are mostly based on grouting methods, which are more suitable for simulating deep soil reinforcement in the case of undisturbed soil samples. In order to simulate shallow soil reinforcement and achieve more uniformly reinforced soil, reinforced soil specimens in this study were fabricated via a mixing-compaction method using cylindrical molds (39.1 mm inner diameter × 80 mm height). The procedure included: (1) weighing a predetermined mass of standard sand, which is calculated based on the relationship between relative density *D*_r_ and void ratio *e* (Equation (1)); (2) uniformly blending the sand with urease solution, cementation solution, and glutinous rice slurry at designated ratios; (3) compacting the mixture into the mold in five layers via a metal rammer, with compaction energy values of 25.2 J, 43.2 J, 64.8 J, and 97.2 J applied to soil samples of relative density *D*_r_ = 0.6, 0.7, 0.8, and 0.9; and (4) curing the specimens at 25 °C and 50% relative humidity for 7 days. After demolding, the specimens were oven-dried at 104 °C to a constant mass.

(1) $$D_{r}=\frac{e_{\max}-e}{e_{\max}-e_{\min}}$$

### 2.3. Experimental Design

To investigate the effects of relative density (*D*_r_) and glutinous rice slurry volumetric content (*V*_G_) on the mechanical behavior of sand, triaxial permeability tests (Group A) and unconfined compressive strength (UCS) tests (Group C) were conducted, as shown in Table 1. where the subscripts i (1–4) and j (1–7) denote the sequentially ordered values of *D*_r_ and *V*_G_, respectively, as listed in table. The maximum value of V_G_ for the G-EICP specimen is 50%, ensuring sufficient EICP solution for calcite generation. In order to investigate the contributions of EICP and glutinous rice slurry to combined strengthening, plain soil was set as the reference group (Group B). All samples were tested in triplicate under the same conditions, and the results shown in this study are the average values based on the three tests. The UCS test was conducted at a loading rate of 0.5 mm/min. For the triaxial permeability test, a confining pressure (*σ*_3_) of 50 kPa and a seepage pressure (*p*) of 10 kPa were applied for group B. For group A, σ_3_ was set at 50 kPa and 100 kPa, and p was set at 10 kPa, 30 kPa, 50 kPa, and 70 kPa. The maximum cell pressure was set to 100 kPa, considering the depth ranges associated with the subgrade backfill and shallow foundation. Additionally, scanning electron microscopy (SEM, by Zeiss Sigma300, Oberkochen, Germany), micro-computed tomography (CT, by Zeiss Xradia 520 Versa, Oberkochen, Germany), and Fourier-transform infrared spectroscopy (FTIR, Nicolet iS50, USA) analyses were carried out on selected specimens (Ⅰ, Ⅱ, Ⅲ, Ⅳ, and Ⅴ) to characterize the pore structure evolution and elucidate the microscale reinforcement mechanisms of the G-EICP system.

### 2.4. FTIR Test Processing

FTIR analysis was performed on vacuum-dried samples (105 °C, 24 h) mixed with KBr (1:100 mass ratio) under a nitrogen atmosphere and ground to a particle size of <2 μm. The mixture was pressed into pellets at a pressure of 10 MPa for 2 min. After polystyrene calibration and background subtraction (32 scans, 4 cm^−1^ resolution), spectra were acquired from 4000 to 400 cm^−1^. Data processing included baseline correction, Savitzky-Golay smoothing, and spectral deconvolution using the OMNIC software (Version 9.2), with peak assignments verified against standard databases.

### 2.5. CT Test Processing and Analysis

**Figure 2:**
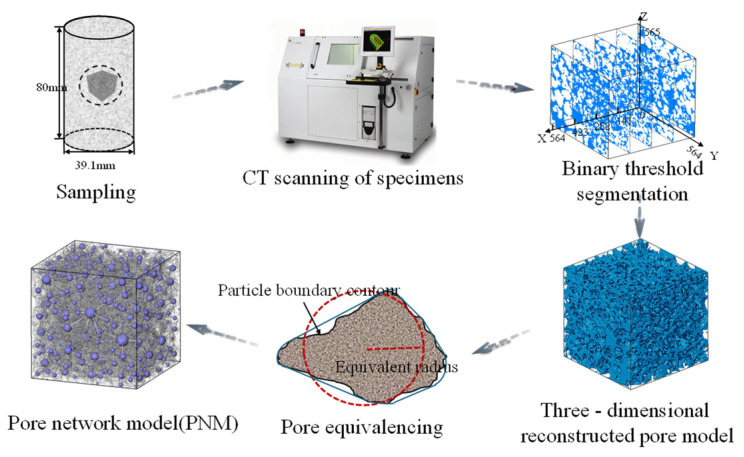
Steps of CT image data processing (the grey lines represent the pore throats in the last steps).

As a naturally porous granular material, the spatial arrangement of sand particles and their pore structures directly influences soil strength and permeability. This study employed micro-computed tomography (micro-CT) imaging coupled with digital image processing techniques to investigate the effects of reinforcement methods on the pore structure of sandy soils. For sample Group Ⅰ to Ⅴ (specified in Table 1), a Zeiss Xradia 520 Versa X-ray micro-CT (by Zeiss, Oberkochen, Germany) system was used to scan the central region of the soil specimen, generating 3D data with a spatial resolution of 17.7108 μm (Figure 2). The processing workflow comprised the following steps:

Image Denoising and Smoothing: noise reduction and edge detection were performed using the Unsharp Masking and Sobel modules in Avizo software (Version 2020.1). This step suppressed the image noise, smoothed the texture discontinuities, and enhanced the grayscale contrast between the soil skeleton and background.

- Contrast Enhancement: The image contrast was optimized to amplify the structural distinctions between the solid particles and air-filled pores, thereby improving phase differentiation for subsequent analyses.
- Threshold Segmentation: The Interactive Threshold module was applied to binarize CT slice images, enabling the precise identification of solid particles and pore spaces.
- 3D Reconstruction: The selected voxel regions were projected onto 2D planes, followed by volumetric stacking to reconstruct the 3D pore architecture of the soil matrix.

Pore Feature Extraction and Visualization: quantitative analysis of the reconstructed 3D pore structure was conducted to extract geometric parameters (e.g., pore size distribution, and connectivity). The results were visualized using a pore network model (PNM), which abstractly represents complex pore geometries as interconnected nodes and throats.

**Figure 3:**
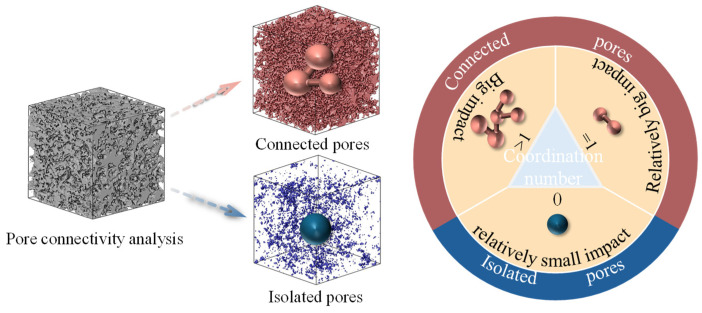
Schematic diagram of pore connectivity versus coordination number.

Soil Soil pores can be classified into connected pores and isolated pores based on their connectivity. Connected pores form interconnected networks that facilitate fluid flow and particle displacement under stress, thereby increasing permeability but reducing the mechanical strength. In contrast, isolated pores, which lack connections to other voids, restrict fluid movement and exhibit minimal influence on both permeability and strength (Figure 3 shows the structural categorization). The coordination number—defined as the number of pores directly connected to a given pore—quantifies the connectivity and spatial topology of the pore networks. A higher coordination number indicates stronger inter-pore linkages and more open structures. Specifically:

- Coordination number > 1: Connected pores adopt a tree-like structure, which is characteristic of branched, high-connectivity networks.
- Coordination number = 1: The pores exhibit a dumbbell-shaped structure, representing linear connections between two adjacent pores.
- Coordination number = 0: Pores exist as isolated entities, contributing negligibly to permeability or strength.

Permeability is governed by both pore development (porosity) and pore-throat architecture. To elucidate the relationship between permeability and pore structure, a systematic classification and characterization of pore-throat features were conducted. Leveraging the pore network model (PNM) derived from soil specimens, binary CT images were segmented into two distinct phases: the pore phase (void space) and matrix phase (soil particles), each assigned specific grayscale values. Individual pores were geometrically represented by embedding equivalent spheres, where the maximum distance within a pore was halved to define the sphere radius. The minimum radius between adjacent pores is designated as the throat radius.

## 3. Results and Discussion

### 3.1. Analysis of Permeability Test Results

This study investigated the effects of four variables—relative density (*D*_r_), glutinous rice slurry volumetric content (*V*_G_), confining pressure (*σ*_3_), and seepage pressure (*p*)—on the permeability coefficient (*k*) of reinforced sand. The influence of each variable is discussed sequentially below.

#### 3.1.1. Effects of D_r_ and V_G_ on Permeability

**Figure 4:**
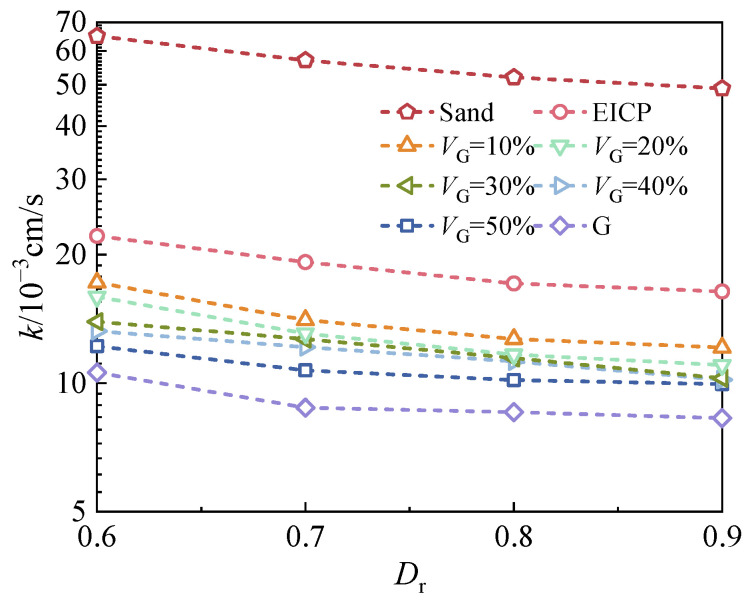
Relationships between permeability coefficient with *D*_r_ and *V*_G_.

Figure 4 illustrates the permeability coefficients of Groups A and B under *σ*_3_ = 50 kPa and *p* = 10 kPa, demonstrating the combined effects of *D*_r_ and *V*_G_. As shown in Figure 4, the permeability coefficient for all samples generally decreases with increasing *D*_r_, as expected. The most significant reduction occurred between *D*_r_ = 0.6 and *D*_r_ = 0.7. Subsequently, the permeability coefficients of all soil samples tended to stabilize, especially for those containing glutinous rice slurry. For glutinous rice slurry reinforced (G) and G-EICP specimens, a sufficient quantity of calcium carbonate and glutinous rice slurry at a lower *D*_r_ can significantly block the pore-throat and reduce permeability.

Furthermore, at a fixed *D*_r_, the permeability coefficient decreased with increasing *V*_G_. A notable reduction of 26.14% in the average permeability was observed when *V*_G_ increased from 0% to 10%. Beyond this threshold, the permeability stabilized, indicating that a modest addition of glutinous rice slurry effectively enhances anti-seepage performance. The authors made the following inferences regarding this phenomenon: the calcium carbonate crystals generated by EICP can block larger pores between soil particles, resulting in a decrease in the permeability coefficient compared to plain sand. After adding 10% glutinous rice slurry, the G-EICP aggregates further block the larger pores, and the smaller pores are also blocked by the glutinous rice slurry; hence, the permeability coefficient decreases further. However, more glutinous rice slurry cannot compensate for the reduced blockage of pores by calcium carbonate; therefore, the permeability coefficient cannot be further reduced.

Based on the discussions above, it can be concluded that the effect of glutinous rice slurry on the permeability coefficient of sandy soil is more pronounced when *D*_r_ is less than 0.7, with the dosage does not exceed 10%

#### 3.1.2. Effects of σ_3_ and P on Permeability

**Figure 5:**
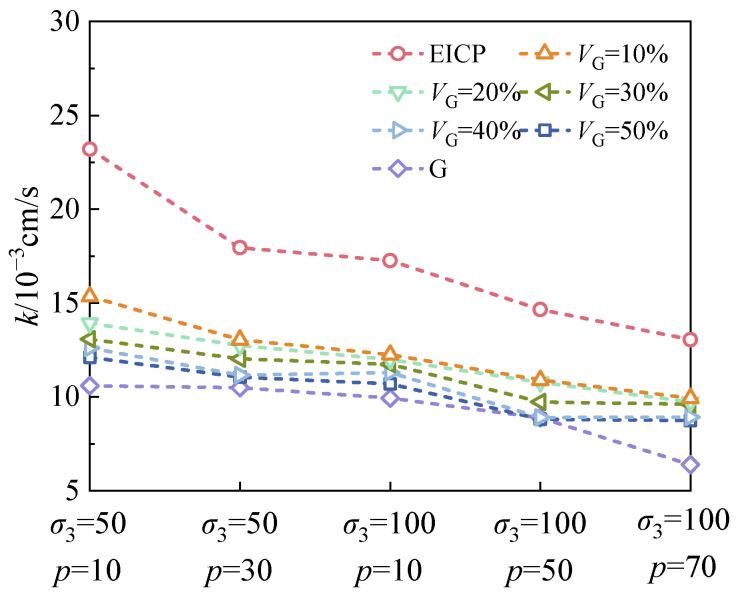
Relationships between permeability coefficient with *σ*_3_ and *p*.

As shown in Figure 4, the glutinous rice slurry and EICP tend to have a more significant impact on the soil’s permeability under conditions of lower density. Therefore, different soil samples with *D*_r_ = 0.6 are used here for further investigation. Figure 5 presents the permeability coefficients of specimens at *D*_r_ = 0.6, highlighting the interplay between cell pressure and *p*. It can be seen that for a given cell pressure, the permeability coefficient slightly decreased with increasing *p*. This phenomenon can be attributed to a dual mechanism: firstly, the increased flow resistance caused by aggregates in the G-EICP-reinforced sand; secondly, a higher *p* can tighten the microstructure of the soil [27], which narrows water migration paths. This trend is also reported in a previous study by our research group [28]. Conversely, at a fixed *p*, a higher cell pressure reduces the permeability coefficient due to the progressive constriction of flow channels under elevated confining stress, thereby limiting the water flux per unit time. Notably, similar trends were observed for G-EICP-reinforced sand with *V*_G_ = 50%; however, a detailed discussion is omitted here for brevity.

### 3.2. Analysis of UCS Test Results

**Figure 6:**
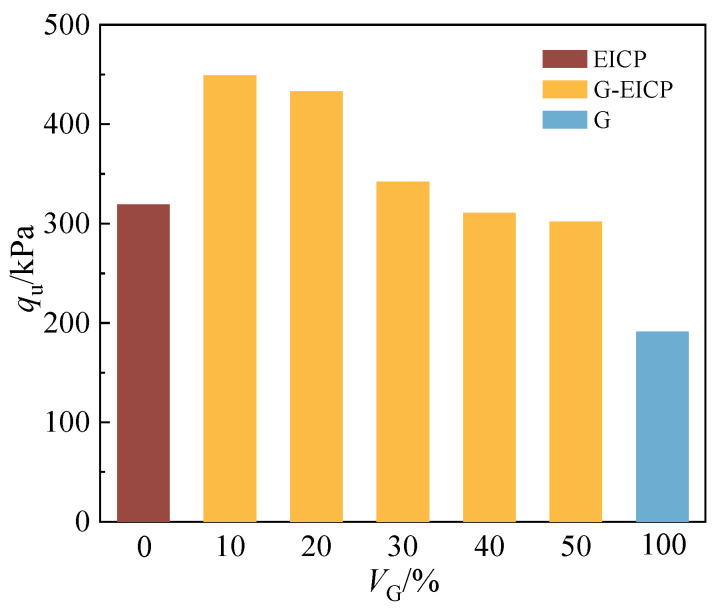
Relationship between UCS of reinforced soil with *V_G_.*

Figure 6 illustrates the influence of varying *V*_G_ on the strength of reinforced soils at a *D*_r_ of 0.6. The reason for choosing soil with *D*_r_ of 0.6 is the same as that mentioned in Section 3.1.2. The results reveal a non-linear trend in soil strength with increasing *V*_G_: the strength of the reinforced soil first increases and then decreases. At low *V*_G_ levels (0–10%), the strength increases significantly, reaching a maximum value of 449.2 kPa. When the *V*_G_ increased from 10% to 50%, however, the strength of the reinforced soil exhibited a progressive decline, decreasing by −3.61%, −21.00%, −9.24%, and −2.77% respectively compared to the previous levels. Notably, soil treated solely with glutinous rice slurry exhibits lower strength compared to EICP-treated soil. In contrast, the combined use of EICP and glutinous rice slurry markedly enhances strength, peaking at *V*_G_ = 10% in the scale of this study. This phenomenon can be attributed to the synergistic interaction between the glutinous rice slurry and calcium carbonate. The authors propose the following hypothesis: at lower *V*_G_ levels, the nucleation-promoting effect of the slurry becomes pronounced, leading to localized aggregation of calcium carbonate at slurry-distributed zones, thereby enhancing soil strength. However, when *V*_G_ exceeds an optimal value, the limited spatial distribution of calcium carbonate precipitation and over-reliance on amylopectin-mediated cohesion result in a decline in soil strength. This trend is further explained in Section 3.3. At lower *V*_G_ levels (0–10%), the slurry effectively fills the soil pores, while calcium carbonate enhances interparticle bonding via nucleation effects, thereby improving strength. However, at higher *V*_G_ ratios, the cohesive contribution of amylopectin, the primary component of the glutinous rice slurry, becomes insufficient to counteract the reduced calcium carbonate aggregation, leading to diminished strength.

When integrating the permeability and strength test results, it is evident that the impermeability of the G-EICP-reinforced soil increases continuously with increasing *V*_G_, whereas the strength follows an initial increase, followed by a decline. This indicates that the glutinous rice slurry contributes more significantly to enhancing impermeability than strength. Additionally, as an organic material, glutinous rice slurry is susceptible to biodegradation when used independently. However, at moderate *V*_G_ levels (10%), calcium carbonate generated by EICP establishes an alkaline environment (pH = 8.3 in this study), which has been reported to mitigate organic degradation [29], while simultaneously improving both strength and impermeability. Based on these findings, a glutinous rice slurry optimal volume ratio of10%is recommended. Within this range, the synergistic reinforcement effect of EICP and glutinous rice slurry achieves a balance, maximizing the strength and impermeability enhancements while avoiding the strength reduction caused by excessive slurry content.

### 3.3. Analysis of Micro- Mechanism of G-EIC- Reinforced Soil

#### 3.3.1. SEM Results Analysis

**Figure 7:**
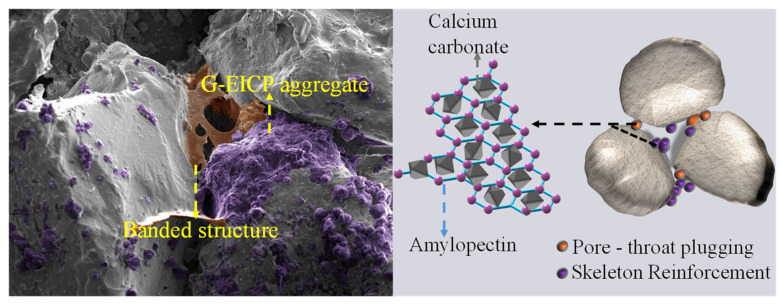
SEM and potential reinforcement mechanisms diagram of G-EICP-reinforced soil.

To further elucidate the mechanism underlying the macroscopic behavior of G-EICP-reinforced sand, the microstructural characteristics of treated soils were analyzed through scanning electron microscopy (SEM). In the EICP process, calcium carbonate primarily reinforces the soil through cementation of soil particles. The fluidic nature of the glutinous rice slurry allows it to be distributed extensively among soil particles as flakes or fine particles, effectively filling interparticle pores. Figure 7 shows a 500× magnification SEM image of the G-EICP-reinforced soil (*V*_G_ = 10%). Calcium carbonate crystals formed larger G-EICP aggregates by bonding with glutinous rice slurry, which filled the pores between soil particles, indicating that the glutinous rice slurry facilitates nucleation sites for calcium carbonate precipitation. In major pore regions, these aggregates act as binding agents, enhancing soil integrity by cementing particles together. Concurrently, the G-EICP aggregates on the surface of soil particles may increase the surface friction of soil particles, thereby enhancing the strength of the soil (which requires further experimental verification). Based on the SEM observations and macroscopic experimental patterns, the authors propose the following hypotheses regarding the reinforcement mechanisms: (1) skeletal reinforcement: the extended amylopectin network structure can combine well with the calcium carbonate crystals distributed in the soil, thereby aggregating into larger G-EICP aggregates. These aggregates can effectively connect soil particles and play a role in enhancing the framework. This effect is mainly reflected in the increase in soil strength. (2) pore-throat clogging: The ribbon-like structures of the glutinous rice slurry formed in the pore-throat parts can effectively block the interparticle pore-throats, thereby reducing pore connectivity. This effect is mainly reflected in the reduction of the soil permeability coefficient.

#### 3.3.2. FTIR Results Analysis

**Figure 8:**
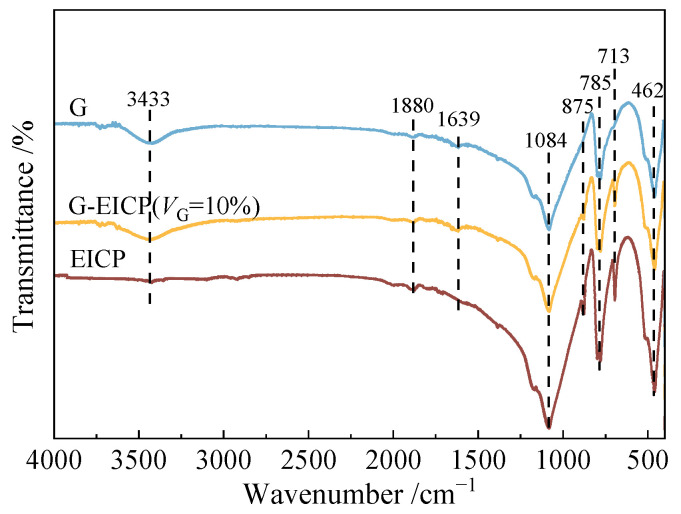
FTIR test results of different reinforced soil specimens.

To evaluate the chemical composition and micro-mechanism of sand reinforced with EICP, G-EICP, and G, Fourier-transform infrared spectroscopy (FTIR) analysis was performed on different samples, and the results are shown in Figure 8. It can be seen that the characteristic peaks at 462, 785, and 875 cm^−1^ correspond to silicate groups (Si-O-Si), where the 785 cm^−1^ peak represents the symmetric stretching vibration of quartz, and the 1084 cm^−1^ peak arises from the superposition of residual Si-O vibrations and polysaccharide C-O stretching vibrations. Upon introducing glutinous rice slurry (G), the intensity of silicate peaks attenuates, indicating that organic components suppress the infrared response of sand particles through a surface coating effect. In EICP and G-EICP-reinforced specimens, sharp absorption peaks at 713 cm^−1^ and 875 cm^−1^ match the characteristic spectrum of calcite-type calcium carbonate [30], confirming the formation of calcite via EICP reactions. Additionally, the broad peak at 3343 cm^−1^ observed in the G-EICP and G samples can be attributed to the O-H stretching vibration of the amylopectin present in the glutinous rice slurry. The above results indicate that the main mechanism of glutinous rice slurry for soil reinforcement is as follows: the O-H groups in its polysaccharide components enhance the connections between sand particles, thereby improving the overall strength of the sand, and the coating effect of the glutinous rice slurry also increases the interfacial friction between particles. The G-EICP method achieves its reinforcing effect through the mechanisms mentioned above and the cementation of calcium carbonate crystals.

### 3.4. Analysis of 3D Pore Structure of G-EICP-Reinforced Soil

#### 3.4.1. Analysis of Pore Structure Results

**Figure 9:**
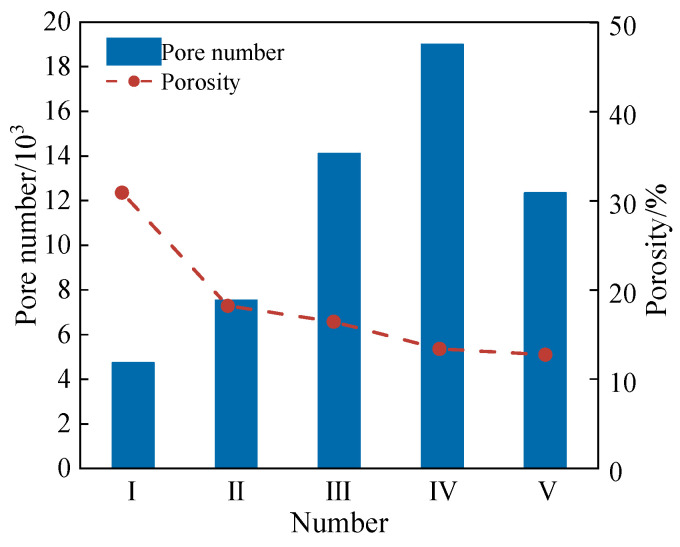
Number of pores and porosity of sample Ⅰ (Plain sand), Ⅱ (EICP), Ⅲ (G-EICP, *V*_G_ = 10%), Ⅳ(G-EICP, *V*_G_ = 50%) and Ⅴ (G).

**Figure 10:**
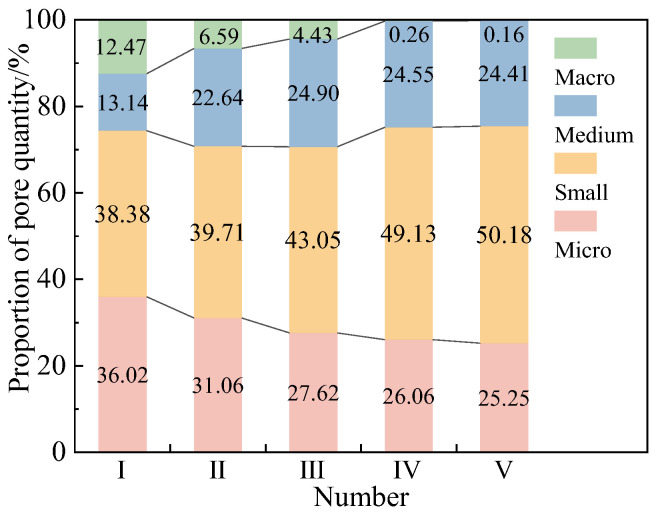
Percentage of pore size distribution for sample Ⅰ (Plain sand), Ⅱ (EICP), Ⅲ (G-EICP, *V*_G_ = 10%), Ⅳ (G-EICP, *V*_G_ = 50%) and Ⅴ (G).

Based on CT scanning data, soil pores were classified into four categories according to the equivalent radius: micropores (0–40 μm), small pores (40–100 μm), medium pores (100–500 μm), and macropores (>500 μm). The statistical distributions of the pore characteristics of all the soil samples are shown in Figure 9 and Figure 10. As shown in Figure 9, the porosities of all the reinforced soils decreased compared to that of untreated soil (Sample I). Notably, the porosity of the G-reinforced soil (Sample V) was lower than that of the EICP-reinforced soil (Sample II), indicating that the glutinous rice slurry exhibits superior pore-filling efficiency compared to EICP alone. For Samples II to IV, the rate of porosity reduction gradually diminished with increasing *V*_G_. This observation aligns with the permeability test results, as the incorporation of glutinous rice slurry effectively fills interparticle voids, reduces hydraulic pathways, and thereby decreases permeability. Regarding pore quantity, all reinforced soils exhibited an overall increase in the total pore number, with a trend of an initial increase followed by a decline as the *V*_G_ increased.

Figure 10 reveals that a higher *V*_G_ led to a progressive reduction in the proportion of macropores and micropores, accompanied by an increase in the medium pore fraction. Meanwhile, the proportion of small pores stabilized at approximately 50% across all reinforced samples. These shifts in the soil’s pore size distribution are attributed to the addition of glutinous rice slurry, as the G-EICP aggregates transform large and mesopores into multiple small and micropores. As the glutinous rice slurry content increases, these small and micropores are further filled, thereby enhancing the compactness of the sand.

Incorporating the discussions above and macro test results, the synergistic effect of glutinous rice slurry and calcium carbonate can effectively fill soil pores, forming a stable skeletal structure. This action reduces the pore connectivity to some extent, helps distribute external hydraulic loads, and suppresses the movement and deformation of soil particles, thereby improving the strength and impermeability of sand.

#### 3.4.2. Analysis of Pore Connectivity

Table 2 presents the pore connectivity characteristics of the different specimens. Significant structural modifications in the pore structure are observed in the reinforced soils compared to the plain soil (Specimen I). With increasing *V*_G_ content (0–100%), both the total pore quantity and the number of isolated pores progressively increases, indicating enhanced discrete flow channels that prolong the fluid retention time within the pore network, consequently reducing the permeability. The connected pore population, however, demonstrates an initial increase, followed by a subsequent reduction, peaking at the specimen with *V*_G_ = 50%. Although the connected pore count decreases in the G-reinforced specimens (*V*_G_ = 100%), it remains substantially higher than those in both plain soil (I) and EICP-treated soil (II), consistent with the pore quantity variation pattern discussed in Section 3.4.1. This suggests that moderate incorporation of glutinous rice slurry effectively optimizes pore connectivity, while excessive *V*_G_ content induces pore structure reorganization and partial pore occlusion, ultimately diminishing connected pores.

Soil failure mechanisms are intrinsically linked to crack propagation, in which connected pores may serve as crack initiation sites. These pores progressively interconnect under stress to form fissures that culminate in structural failure. The coordination number exhibits a marked decrease from 1.858 to 0.249 with increasing *V*_G_, revealing that the addition of glutinous rice slurry reduces the integrity of the inter-pore connection. The originally well-connected pore network undergoes structural densification through pore-filling effects, resulting in a diminished connectivity and higher strength. This deterioration in pore connectivity fundamentally restricts fluid percolation pathways and increases flow resistance, thereby accounting for the reduced permeability coefficients. Furthermore, G-EICP aggregate formation facilitates pore occlusion while enhancing interparticle bonding and adsorption effects, collectively improving soil structural integrity and stability.

#### 3.4.3. Analysis of Pore-Throat

**Figure 11:**
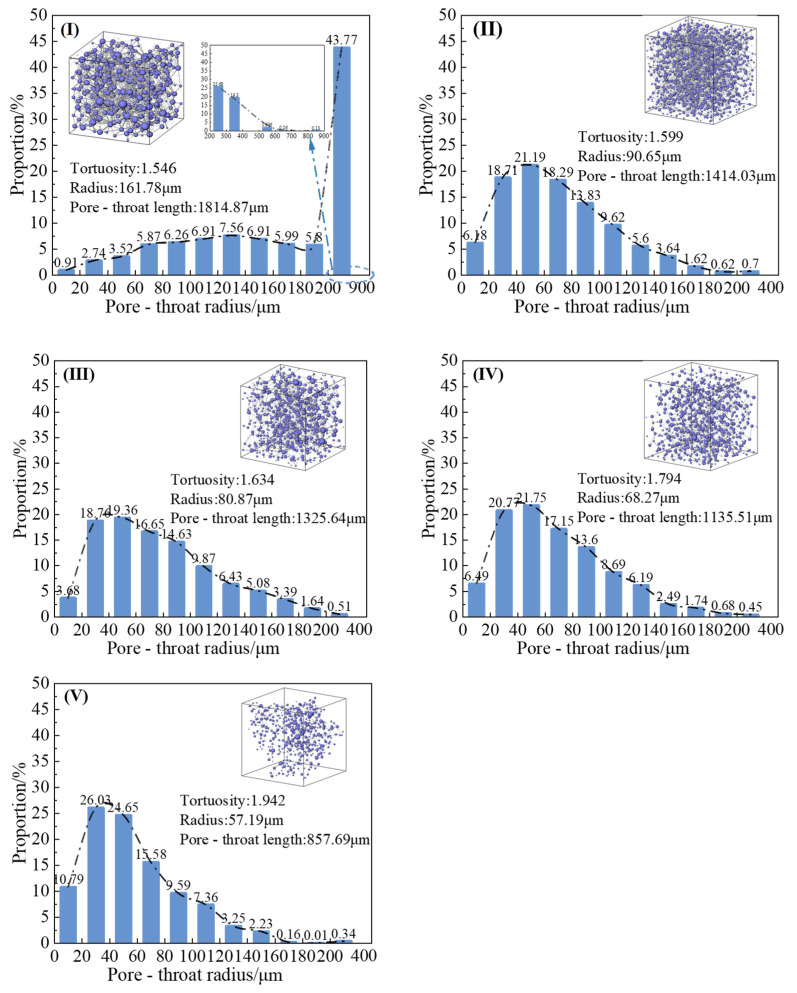
Pore-throat size data and distribution for sample (**Ⅰ**) (Plain sand), (**Ⅱ**) (EICP), (**Ⅲ**) (G-EICP, *V*_G_ = 10%), (**Ⅳ**) (G-EICP, *V*_G_ = 50%) and (**Ⅴ**) (G).

Figure 11 illustrates the key pore-throat parameters, including pore-throat dimensions, pore radii, and tortuosity across all specimens. As shown, the pore-throat dimensions of all soils exhibit continuous normal distributions. The inherent pore structure of sand particles, characterized by granular stacking, is altered by calcium carbonate-glutinous rice slurry aggregates adhering to particle surfaces, which obstruct the original percolation pathways (i.e., pore-throats). With increasing *V*_G_ content (0~100%), both the throat length and radius progressively decrease, resulting in more convoluted pore structures (i.e., elevated tortuosity). While reduced throat lengths marginally shorten fluid migration paths, narrowed throat radii amplify flow resistance by constricting water passageways, collectively contributing to a decrease in permeability coefficients. Enhanced tortuosity further complicates the fluid pathways, intensifying the frictional interactions between the fluid molecules and pore walls, thereby exacerbating the permeability reduction.

Notably, pore-throat radius distribution undergoes significant alterations. The proportion of throat radii within 0~200 μm gradually increases from Specimen I (plain soil) to Specimen V (G-reinforced soil), while the fraction of radii exceeding 200 μm is significantly reduced. In treated soils, throat radii predominantly concentrate within smaller ranges (0~200 μm), indicating a marked reduction in large, unobstructed flow channels and a predominance of finer, labyrinthine pathways. This shift forces fluid transport to increasingly rely on narrow, tortuous pores, severely constraining the water migration capacity and amplifying the hydraulic resistance. Consequently, these microstructural modifications synergistically reduce the permeability coefficients by impeding efficient fluid percolation through the soil matrix.

As shown in Figure 11, the pore-throat size distribution does not conform to the standard normal distribution. Therefore, this study employs the skewness and kurtosis parameters to quantify the characteristics of the pore structure. As indicated in Table 3, the pore-throat radius distribution of the plain sand sample exhibits a strong positive skewness (skewness = 2.5) and high kurtosis (kurtosis = 5.2), indicating the presence of a larger number of pore-throats with relatively larger radii. The skewness of the reinforced soils is significantly reduced compared to that of the plain sand, reflecting a shift in the pore-throat distribution toward smaller radii, dominated by fine pore-throats. This may be attributed to the fact that crystals and glutinous rice slurry are more likely to form blockages in larger pore-throats. Additionally, the kurtosis of the reinforced soils also shows a marked decrease and becomes negative, suggesting the absence of a dominant range of pore-throat radii in the reinforced soils. The reinforcing materials divide the continuous pore-throats into discrete, smaller micropores. The G-EICP-reinforced soil with *V*_G_ = 15% has the smallest skewness and kurtosis values, indicating that at this point, the combined action of glutinous rice slurry and EICP significantly homogenizes the soil’s pore system and disrupts the dominant seepage pathways.

## 4. Conclusions

This study investigated the effects of various factors on the strength and permeability of G-EICP-reinforced sand through a series of unconfined compressive strength tests, triaxial permeability tests, SEM, and micro-CT analyses. The following conclusions were drawn:

1. All reinforced soil specimens exhibited lower permeability coefficients than the untreated soil. The permeability coefficient gradually decreased with increasing relative density (*D*_r_), glutinous rice slurry volume content (*V*_G_), seepage pressure (*p*), and confining pressure (*σ*_3_). Notably, more pronounced variations in permeability were observed during the initial stages of lower *D*_r_ and smaller *V*_G_.
2. Significant differences in permeability and strength characteristics were observed in G-EICP-reinforced soil under varying glutinous rice slurry volume ratios. Specimens with low slurry ratios (*V*_G_ = 0~20%) demonstrated superior strength performance, reaching a maximum unconfined compressive strength of 449.2 kPa at *V*_G_ = 10%. Comparative analysis revealed that EICP-reinforced soil exhibited higher strength than G-reinforced soil when applied separately.
3. Microstructural analysis demonstrated that glutinous rice slurry particles serve as nucleation sites for calcium carbonate precipitation in G-EICP-reinforced soil. This mechanism regulates the morphology, size distribution, and spatial arrangement of calcium carbonate crystals, forming distinctive slurry−calcium carbonate aggregates. These aggregates appear to contribute to soil stability, possibly through two mechanisms: “pore throat clogging” that reduces permeability and “skeleton reinforcement”, which improves structural integrity.
4. All three treatment methods (EICP, G, and G-EICP) effectively reduced soil porosity while modifying the pore size distribution and spatial configuration. With increasing *V*_G_, the treated soil exhibited higher total pore counts, accompanied by reduced connectivity (manifested through decreased numbers of connected pores and coordination numbers) and increased tortuosity. These microstructural modifications collectively contributed to significant improvements in both the mechanical strength and hydraulic characteristics compared to untreated soil.

According to the above conclusions, G-EICP has been proven to enhance the strength and reduce the permeability of sandy soil, which endows it with potential for application in engineering projects. However, the number of parallel specimens in this study for each test was three. Therefore, the experimental conclusions drawn may not be universally applicable, and further extensive experimental research is needed. In addition, current studies on G-EICP-reinforced sand are still in the laboratory stage and focus mainly on short-term effects. Future work should involve large-scale model tests and investigate the long-term behavior of G-EICP-reinforced sand.

## Figures and Tables

**Table 1:** Experimental design in this study.

ID	*D*_r_	*V*_G_(%)	Test Type
A_ij_	0.6, 0.7, 0.8, 0.9	0, 10, 20, 30, 40, 50, 100	Permeability
B_i_	0.6, 0.7, 0.8, 0.9	Plain sand	
C_j_	0.6	0, 10, 20, 30, 40, 50, 100	UCS
Ⅰ	0.6	Plain sand	SEM, micro-CT, FTIR
Ⅱ	0.6	0	
Ⅲ	0.6	10	
Ⅳ	0.6	50	
Ⅴ	0.6	100	

**Table 2:** Pore connectivity and permeability coefficient of sample Ⅰ (Plain sand), Ⅱ (EICP), Ⅲ (G-EICP, *V*_G_ = 10%), Ⅳ(G-EICP, *V*_G_ = 50%) and Ⅴ (G).

Sample ID	Ⅰ	Ⅱ	Ⅲ	Ⅳ	Ⅴ
Pore structure	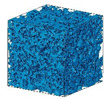	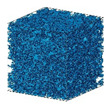	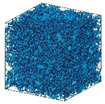	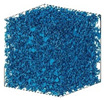	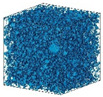
Connected pores PNM	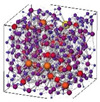	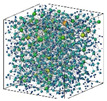	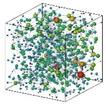	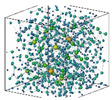	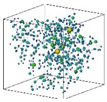
Isolated pores	267	516	709	814	1440
Connected pores	4472	7013	13,395	18,183	10,896
Coordination number	1.858	1.326	0.944	0.353	0.249
*k*(*σ*_3_ = 50, *p* = 10 kPa)	0.065	0.025	0.013	0.012	0.010

**Table 3:** Normalized Skewness and Kurtosis values of pore-throat radius distribution.

Item	Plain Sand	EICP	G-EICP (*V*_G_ = 15%)	G-EICP (*V*_G_ = 50%)	G
Skewness	2.50	0.39	0.32	0.46	0.77
Kurtiosis	5.20	−1.38	−1.47	−1.27	−0.74

## Data Availability

The original contributions presented in the study are included in the article, further inquiries can be directed to the corresponding author.
